# Early Methylglyoxal Exposure Leads to Worsened Cardiovascular Function in Young Rats

**DOI:** 10.3390/nu16132029

**Published:** 2024-06-26

**Authors:** Marcos Divino Ferreira-Junior, Keilah Valéria N. Cavalcante, Jaqueline M. Costa, Amanda S. M. Bessa, Andreia Amaro, Carlos Henrique de Castro, Carlos Henrique Xavier, Sónia Silva, Diogo A. Fonseca, Paulo Matafome, Rodrigo Mello Gomes

**Affiliations:** 1 Coimbra Institute for Clinical and Biomedical Research (iCBR), Faculty of Medicine, University of Coimbra, 3000-548 Coimbra, Portugalandreia.amaro15@hotmail.com (A.A.); sonias@ci.uc.pt (S.S.); diogo.fonseca@ff.uc.pt (D.A.F.); 2 Department of Physiological Sciences, Universidade Federal de Goiás, 74690-900 Goiás, Brazil; jaquelinemoura2012@hotmail.com (J.M.C.); amandabessa1@gmail.com (A.S.M.B.); castro@ufg.br (C.H.d.C.); carlosxavier@ufg.br (C.H.X.); gomesrm@ufg.br (R.M.G.); 3 Clinical and Academic Centre of Coimbra (CACC), 3004-531 Coimbra, Portugal; 4 Center for Innovative Biomedicine and Biotechnology (CIBB), University of Coimbra, 3000-548 Coimbra, Portugal; 5 Faculty of Pharmacy, University of Coimbra, 3000-548 Coimbra, Portugal; 6 Coimbra Health School (ESTeSC), Polytechnic University of Coimbra, 3045-043 Coimbra, Portugal

**Keywords:** glycotoxins, methylglyoxal, BBGC, lactation, left ventricular dysfunction

## Abstract

Background: Though maternal diabetes effects are well described in the literature, the effects of maternal diabetes in postnatal phases are often overlooked. Diabetic individuals have higher levels of circulating glycotoxins, and there is a positive correlation between maternal-derived glycotoxins and circulating glycotoxins in their progeny. Previous studies evaluated the metabolic effects of high glycotoxin exposure during lactation in adult animals. However, here we focus on the cardiovascular system of juvenile rats. Methods: For this, we used two experimental models: 1. High Methylglyoxal (MG) environment: pregnant Wistar rats were injected with PBS (VEH group) or Methylglyoxal (MG group; 60 mg/kg/day; orally, postnatal day (PND) 3 to PND14). 2. GLO-1 inhibition: pregnant Wistar rats were injected with dimethyl sulfoxide (VEH group) or a GLO-1 inhibitor (BBGC group; 5 mg/kg/day; subcutaneously, PND1–PND5). The offspring were evaluated at PND45. Results: MG offspring presented cardiac dysfunction and subtly worsened vasomotor responses in the presence of perivascular adipose tissue, without morphological alterations. In addition, an endogenous increase in maternal glycotoxins impacts offspring vasomotricity due to impaired redox status. Conclusions: Our data suggest that early glycotoxin exposure led to cardiac and vascular impairments, which may increase the risk for developing cardiovascular diseases later in life.

## 1. Introduction

The number of diabetic or obese women of childbearing age is increasing, with the respective prevalence projected to increase by almost 10% for diabetes and 57% for obesity or overweight [1,2]. Some of the factors related to higher birth weight are the increased nutritional supply due to the maternal lifestyle and the increased energy available to the foetus due to hyperglycaemia, combined with increased plasma insulin. A classic example of this impact is that children born from mothers with diabetes or with obesity tend to be large for gestational age (LGA) [3,4].

It is also known that due to higher glycemia, diabetic individuals have higher levels of circulating glycotoxins, derived from non-canonical or incomplete glucose metabolism [5]. One of the main glycotoxins is methylglyoxal (MG), which has its main mechanism of action in the formation of adducts with other molecules, called Advanced Glycation End-products (AGEs) [5,6]. The link between diabetes-associated comorbidities and AGEs is well described, and the major end-organ injuries are associated with oxidative stress and the glycation of important proteins [7,8,9,10,11]. In addition to oxidative stress, methylglyoxal-induced cardiac injury is mainly associated with decreased mitochondrial function, even in the absence of hyperglycaemia [9,10,11,12,13,14].

Also important to cardiovascular system health, endothelial function is worsened in cells exposed to MG [11,15,16,17]. Lee and colleagues [16] demonstrated that MG-induced endothelial dysfunction acts via increased oxidative stress and lower autophagy, which leads to apoptosis. Additionally, the perivascular adipose tissue (PVAT), which surrounds large blood vessels, has proven to regulate endothelial function and to change its regulatory effect upon glycotoxin exposure [17,18].

The influence of maternal diabetes on the health of the progeny at adulthood is the aim of several studies and also the aim of the DOHaD (Developmental Origins of Health and Disease) concept. Gestational diabetes is of great importance due to its association with high gestational weight at birth and adverse health outcomes in adulthood [19,20]. Maternal diabetes increases both cardiomyocyte apoptosis and macrophage infiltration in the heart of the offspring [21]. Also, diabetes during the pregnancy leads to impaired mitochondrial dynamism in the offspring, which implies lower adaptation to environmental nutrient changes [22]. In addition, exposure of the foetus/neonate to metabolites via the placenta or breast milk, such as glycotoxins, has been linked to short- and long-term disorders in their development [23,24,25].

It is well known that mother and foetus share nutrients, metabolites, and other biomolecules. Moreover, it has also been proven that maternal glycotoxins are shared with the newborn through the blood [26,27]. However, the important period of lactation is often overlooked. In this sense, Francisco and colleagues [7,25] previously showed that early exposure to MG during the lactation period can induce metabolic disturbances at adulthood. The authors also shown that MG, or at least advanced glycation end-products, were delivered through breast milk, since both direct exposure through injection to the offspring and indirect exposure through oral administration to the mother led to insulin resistance [7,25]. Furthermore, it has been shown that early glycotoxin exposure, induced by the inhibition of Glyoxalase-1 (GLO-1) with BBGC (S-p-Bromobenzylglutathione cyclopentyl diester), leads to decreased milk antioxidant capacity, causes sex-specific neurodevelopmental changes, and results in increased glycation in the visceral adipose tissue in juvenile rats [28,29].

However, despite higher incidence of cardiovascular disease and the knowledge about the effects of acute glycotoxin exposure, little is known about the effects of glycotoxin exposure during early life on the cardiovascular system. Thus, the aim of this study is to evaluate the effects of early glycotoxin exposure on the cardiovascular system of juvenile rats. The main hypothesis is that early glycotoxin exposure leads to cardiovascular impairments due to the accumulation of glycotoxins in the developing heart, changes in PVAT effects on vascular function, and impaired redox state in the vascular endothelium.

## 2. Materials and Methods

All experimental protocols were approved by the Ethics Committee on the Use of Animals of the Federal University of Goiás (007/21) and by the Animal Welfare Committee of the Coimbra Institute for Clinical and Biomedical Research (iCBR), Faculty of Medicine, University of Coimbra (ORBEA 13/2018), and followed both the current legal standards and the ARRIVE guidelines.

### 2.1. Animal Models

Male and female 70-day-old virgin Wistar rats from the Centre for Production and Science in Biomodels (CPCBio/UFG) or from the animal facility of the Institute of Clinical and Biomedical Research of Coimbra (iCBR/FMUC) were housed in the respective animal facilities in the holding institutions. Throughout the experimental period, the animals remained in polypropylene cages (45 × 30 × 15 cm) under controlled conditions of luminosity (12/12 h—lights on 07:00) and temperature (23 ± 2 °C). Mating was performed at a ratio of 2 females to 1 male. Pregnancy was confirmed in the presence of vaginal plug, and females were allocated to individual cages.

#### 2.1.1. Protocol 1—Early Glycotoxin Exposure through Breast Milk

**Figure 1:**
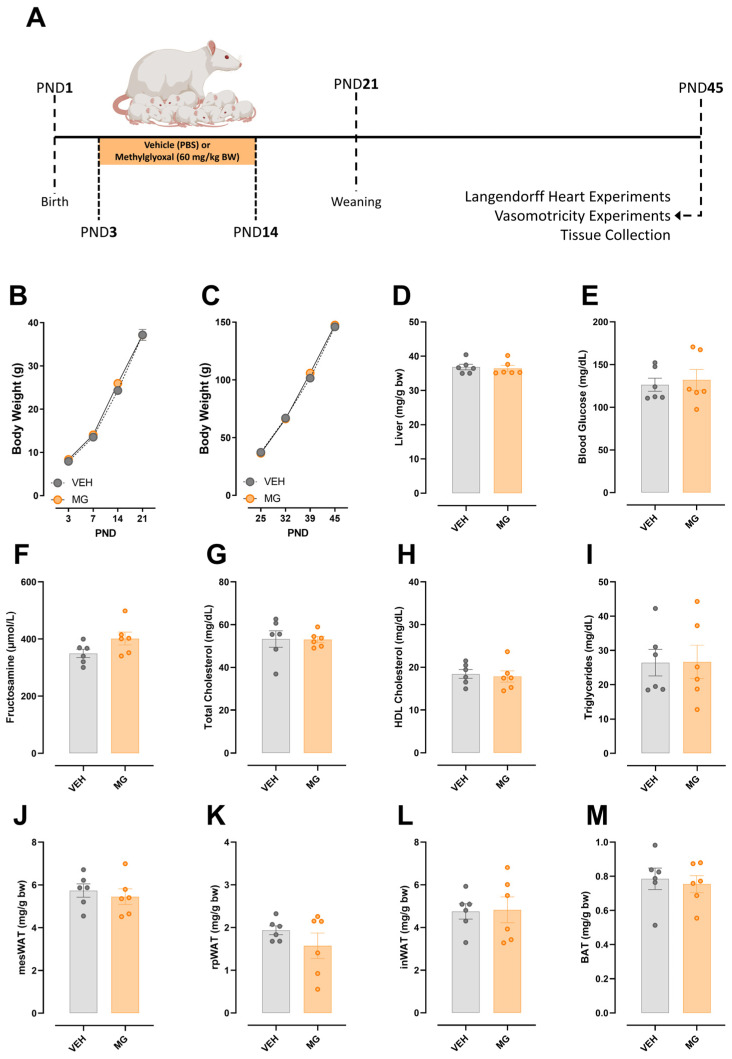
Effects of early methylglyoxal exposure during lactation on the phenotype of young offspring. Experimental design (**A**). Body weight of VEH and MG offspring remains the same before (**B**) and after weaning (**C**). The weight of the most affected tissue, the liver, in MG-injected animals was the same in MG and VEH animals (**D**). Early MG exposure was not able to impact plasma biochemical parameters, namely blood glucose (**E**), fructosamine (**F**), total (**G**) and HDL (**H**) cholesterol, and triglycerides (**I**). The weight of mesenteric (**J**), retroperitoneal (**K**), and inguinal (**L**) white adipose tissues was the same between the groups. In the same sense, the weight of interscapular brown adipose tissue (**M**) was similar between the groups. Two-way ANOVA followed by Sidak’s post hoc test was used for the analysis of time-dependent parameters. Student’s *t*-test was used for the analysis of time-independent parameters.

Pregnant Wistar rats were separated into two groups: VEH (n = 6), in which the vehicle (PBS) was administered orally; and MG (n = 6), in which methylglyoxal was administered orally (60 mg/kg). The treatment was carried out from postnatal day (PND) 3 to PND14. Figure 1A represents the experimental design of the Protocol 1.

At PND45, the end of the experimental period, a batch of animals was euthanized by decapitation for Langendorff heart and vasomotricity experiments.

Another batch of animals was anesthetised with Sodium Thiopental (Cristália, São Paulo, Brazil) for blood collection by puncturing the vena cava. The heart, liver, interscapular brown adipose tissue, and white adipose tissue stocks (mesenteric, retroperitoneal, and inguinal) were dissected and weighed. An intermediate part of the ventricles and a fragment of the thoracic aorta were dissected for further histological analysis.

#### 2.1.2. Protocol 2—Early Glycotoxin Exposure through Maternal GLO-1 Inhibition during Lactation

Pregnant Wistar rats were separated into two groups: VEH (n = 6), in which the vehicle (Dimethyl sulfoxide) was administered intraperitoneally; and BBGC (n = 6), in which BBGC was administered subcutaneously (5 mg/kg). The maximum volume injected was 80 μL. The treatment was carried out from PND1 to PND5. Figure 5A represents the experimental design of the Protocol 2. At PND45, the end of the experimental period, the animals were euthanized by cervical dislocation after anesthesia for vasomotricity experiments, and the heart and the liver were dissected and weighed.

### 2.2. Plasma Biochemical Measurements

From the blood samples of protocol 1, plasma levels of Blood Glucose, Fructosamine, Total and HDL cholesterol, and Triglycerides were measured by enzymatic-colorimetric methods (Bioclin, Belo Horizonte, Minas Gerais, Brazil) with commercial kits, following the manufacturer’s information. The results, except for fructosamine which is expressed in μmol/L, were expressed as mg/dL.

### 2.3. Ex Vivo Experiments

#### 2.3.1. Langendorff Heart

Animals from protocol 1 (n = 6 animals from different litters per group) were decapitated, and the hearts were perfused according to Langendorff technique. After trunk opening, the heart was excised and perfused through the aortic stump with Krebs–Ringer solution (118 mM NaCl, 4.7 mM KCl, 1.25 mM CaCl_2_·2H_2_O, 1.20 mM MgSO_4_·7H_2_O, 1.20 mM KH_2_PO_4_, 26.5 mM NaHCO_3_, and 11.7 mM glucose) at 37 °C with constant oxygenation (5% CO_2_ and 95% O_2_). A water-filled balloon was inserted into the left ventricle and connected to a pressure transducer coupled to a data acquisition system (DataQ Instruments, Akron, Ohio, USA), to acquire intraventricular pressure during the systole (IVSP) and diastole (IVDP). The maximal rate of left ventricular pressure rise (max dP/dt) and maximal rate of left ventricular pressure decline (min dP/dt) were calculated from IVP. The AUC of all parameters was calculated from pre- and post-ischemia periods. The perfusion flow was adjusted to keep the perfusion pressure between 60 and 110 mmHg. The perfusion pressure was monitored through a transducer connected in parallel to the perfusion system. The sample rate was 1 KHz, and the data passed through a low-pass filter of 50 Hz to minimize interferences from the power source. After a basal period (30 to 40 min), the hearts were perfused for an additional 15 min with Krebs–Ringer solution to IVP, Maximum and Minimum dP/dt assessment. Then, the circumflex artery was ligated using a silk suture, and local ischemia was maintained for 30 min. After suture removal, the hearts were normally perfused for another 30 min to assess post-ischemia responses. The results of IVP were expressed in mmHg, the results of dP/dt were expressed in mmHg/s, and the results of AUC were expressed in arbitrary units.

#### 2.3.2. Isolated Aorta Rings

Thoracic aorta rings from the offspring of protocol 1 (4 mm), from animals used in the isolated heart experiment, were placed in 10 mL organ baths at 37 °C containing gassed (95% O_2_ and 5% CO_2_) Krebs–Henseleit solution (118 mM NaCl, 4.6 mM KCl, 3.3 mM CaCl_2_·2H_2_O, 2.4 mM MgSO_4_·7H_2_O, 0.9 mM KH_2_PO_4_, 24.9 mM NaHCO_3_, and 11.1 mM Glucose). The set of rings was assessed as follows: PVAT+ E+; PVAT+ E-; PVAT- E+; and PVAT- E-. Isometric force was evaluated using a force transducer connected to a data acquisition system (DataQ Instruments, USA). After preparation, the rings were initially stretched until the resting tension reached 1.5 g and allowed to equilibrate for 1 h. Endothelium-dependent relaxation was performed with Acetylcholine (ACh; 10 μM) after pre-contraction with Phenylephrine (PHE; 0.1 μM). After the viability test, after a 30 min period for the stabilization of the preparation and exchange of nutrient solution, a pre-contraction of aorta rings using phenylephrine was performed (PHE; 0.1 μM). Then, the E+ rings were submitted to the concentration curve of acetylcholine (ACh; −8; −7.5; −7; −6.5; −6; −5.5; and −5 Log mol/L), and the E- rings were submitted to the concentration curve of Sodium Nitroprusside (SNP; −11; −10.5; −10; −9.5; −9; −8.5; −8; −7.5; −7; −6.5; −6; −5.5; and −5 Log mol/L). Relaxation responses were analysed individually. Following the evaluation of relaxation, after a new period of 30 min for stabilization of the preparation and exchange of the nutrient solution, E-contractile response was assessed thought a concentration curve of PHE (−9; −8.5; −8; 7.5; −7; −6.5; −6; −5.5; and −5 Log mol/L).

Thoracic aorta rings from the offspring of protocol 2 (n = 6 animals from different litters per group) were also placed in 10 mL organ baths at 37 °C containing gassed (95% O_2_ and 5% CO_2_) Krebs–Henseleit solution. Isometric force was evaluated using a force transducer connected to a data acquisition system (PowerLab Instruments, USA). After preparation, the rings were initially stretched until the resting tension reached 1.5 g and allowed to equilibrate for 1 h 30 min. Endothelium-dependent relaxation was performed with ACh after pre-contraction with Noradrenaline (NA; 10 μM). Acetylcholine-induced relaxation was evaluated by a dose–response curve (ACh; −8; −7.5; −7; −6.5; −6; −5.5; and −5 Log mol/L). After the first dose–response curve, a 30-min period for the stabilization of the preparation was performed. Then, the rings were incubated for 30 min with antioxidant (Ascorbic Acid; 100 μM), and another dose–response curve of ACh was performed.

### 2.4. Morphological Analysis

Samples of the heart and aorta (n = 6 animals from different litters per group) were rapidly immersed in KCl (0.1 M), fixed in formalin solution (10%), dehydrated in a series of increasing alcohol concentrations (70% to 100%), diaphanized in xylene, and embedded in histological paraffin. Subsequently, the materials were sectioned in a microtome (RM2245, Leica Microsystems, Wetzlar, Germany) in non-serial cuts of 6 µm thickness that were placed on glass slides and dried in an oven at 37 °C for subsequent staining with Haematoxylin and Eosin (HE; heart and aorta) or Picrosirius-red (PSR; heart).

Photomicrographs were taken using a light microscope coupled to a digital camera (DM500 + ICC50 HD, Leica Microsystems, Wetzlar, Germany) at 1000× magnification to measure left ventricular cardiomyocytes (n = 60/group; HE), at 400× to measure aortic intima-media thickness (n = 30/group; HE) and perivascular fibrosis (n = 30/group; PSR), and at 100× to measure interstitial fibrosis (n = 60/group; PSR). All the experiments were conducted in a blinded way.

#### 2.4.1. Measurement of Cardiomyocyte Diameter and Intima–Media Thickness in Aortas

Photomicrographs of cross-sections of left ventricular cardiomyocytes and thoracic aortas were analysed. The distance between the upper and lower parts of the membrane was measured at the height of the nucleus of each cardiomyocyte. Measurements were made at 4 different points in the middle layer of each slice, in three different slices separated at least by 300 μm in length, to assess the thickness of the intima-media layer. The mean of cardiomyocyte diameter and aortic intima-media thickness for each animal were calculated, and the results were compared between groups.

#### 2.4.2. Perivascular Fibrosis Measurement

Photomicrographs containing collagen markings in fields where arterioles were observed in cross-sections were analysed manually with the ICY software (Institut Pasteur, Paris, France. http://icy.bioimageanalysis.org/, accessed on 10 February 2024). The area of perivascular fibrosis was determined, divided by the area of the lumen of the vessel, resulting in the perivascular fibrosis index, expressed in arbitrary units. The mean of the perivascular fibrosis for each animal was calculated, and the results were compared between groups.

#### 2.4.3. Interstitial Fibrosis Measurement

Interstitial fibrosis was analysed by macro processing using FIJI (ImageJ v1.54), adapted from Hadi and colleagues [30]. Briefly, the images were processed using colour deconvolution to split the picrosirius red staining component from other channels. The picrosirius red channel was submitted to the threshold for evidence of real staining areas. Then, marked areas were quantified. The percentage of interstitial fibrosis was estimated by the ratio between the marked areas and total area of the image. The mean of the percentage of Interstitial Fibrosis was calculated for each animal, and the results were compared between groups.

### 2.5. Statistical Analysis

Sample size was calculated a priori using G*Power (v3.1). Our first outcome was to detect a difference in ex vivo cardiac contractility, since the model used in the protocol 1 was already validated in the study of Francisco and colleagues [25]. For this, a minimum variation of 15% in IVSP was expected. For a power of 80% and a significance level of 5%, this allows detection of a difference of 20% between the groups, with a minimum sample size of six animals per group. For protocol 2, we assumed the same sample size in order to keep the models comparable in statistical terms.

Data are expressed as Mean ± Standard Error of Mean (M ± SEM). Before the use of parametric tests, a normality test (Shapiro–Wilk) was performed. Two-way ANOVA followed by Sidak’s post hoc test was used for time-dependent and before/after analyses (e.g., body weight evolution, evaluation of cardiac function pre- and post-ischaemia). Student’s *t*-test was used for the analysis of time-independent parameters. The significance level was set at *p* < 0.05. For the analyses and graphical representation, GraphPad Prism (v 9.01; GraphPad, San Diego, CA, USA) was used.

## 3. Results

### 3.1. Effects of Early Methylglyoxal Exposure during Lactation on the Phenotype of Young Offspring

Exposure to glycotoxins during lactation does not impact body weight gain during lactation (Figure 1B) or after weaning (Figure 1C). In addition, liver weight (Figure 1D), blood glucose (Figure 1E), fructosamine (Figure 1F), total and HDL cholesterol (Figure 1G,H), triglycerides (Figure 1I), and adipose tissue fat pads (Figure 1J,M) were similar in both groups.

### 3.2. Effects of Early Methylglyoxal Exposure during Lactation on Left Ventricular Cardiac Function

**Figure 2:**
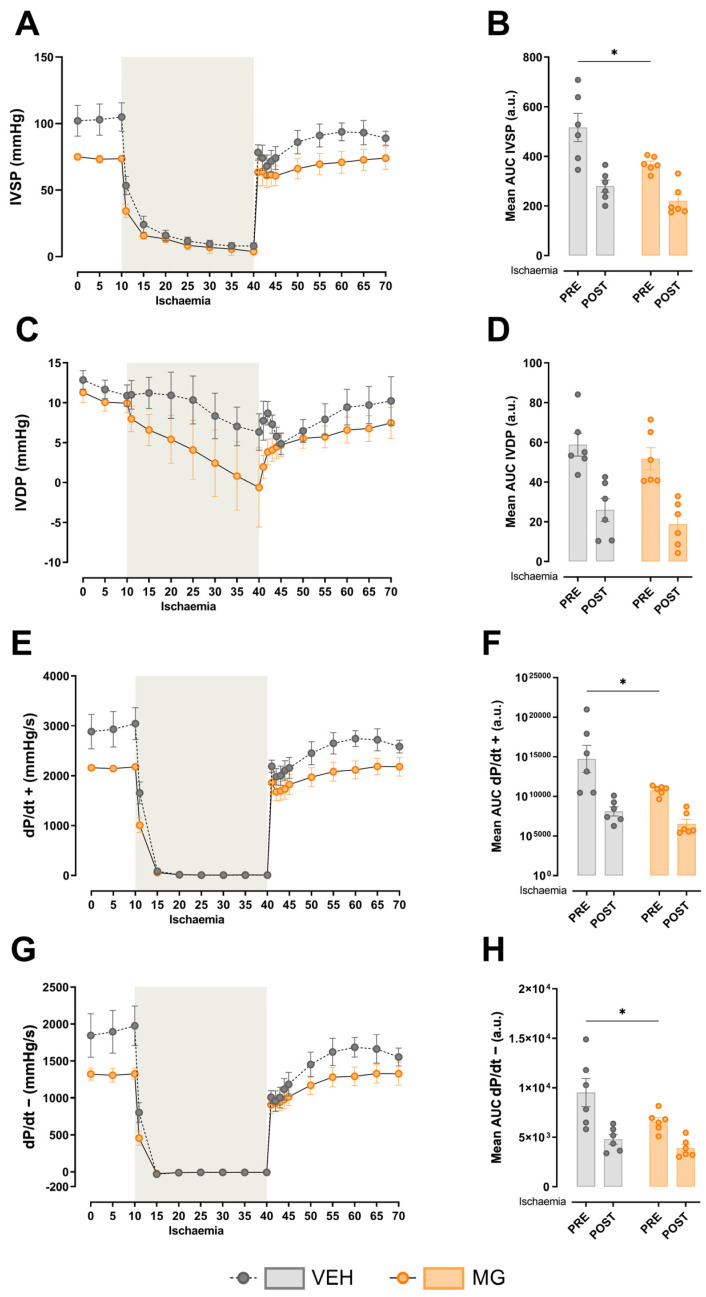
Effects of early methylglyoxal exposure during lactation on left ventricular cardiac function. During ex vivo assessment of basal left ventricular intraventricular pressure, MG offspring developed lower systolic pressure (**A**,**B**), despite no changes in diastolic pressure (**C**,**D**). However, MG hearts also developed lower positive (**E**,**F**) and negative (**G**,**H**) dP/dt. No changes were observed in all evaluated parameters in response to local ischemia promoted by circumflex artery ligature. Two-way ANOVA followed by Sidak’s post hoc test was used for the analysis of pre-/post-ischaemia data. * *p* < 0.05 vs. VEH.

Regarding cardiac function, early exposure to glycotoxins impacts the left ventricular contractility. Our results show that hearts from MG-exposed offspring developed lower intraventricular systolic pressure (Pre-Ischaemia; VEH 516.30 ± 56.89 mmHg vs. MG 368.83 ± 12.48 mmHg; Figure 2A,B; *p* < 0.05), although no changes were observed after local ischaemia. No changes were observed in intraventricular diastolic pressure (Figure 2C,D). As a consequence of lower developed IVSP, both positive and negative dP/dt were different before local ischaemia induced by circumflex artery ligation (dP/dt+ Pre-Ischaemia; VEH 14736.63 ± 1716.83 mmHg/s vs. MG 10780.65 ± 253.37 mmHg/s; *p* < 0.05) (dP/dt− Pre-Ischaemia; VEH 9514.79 ± 1419.16 mmHg/s vs. MG 6577.72 ± 420.11 mmHg/s; *p* < 0.05).

### 3.3. Effects of Early Exposure to Methylglyoxal during Lactation on Vasomotricity

**Figure 3:**
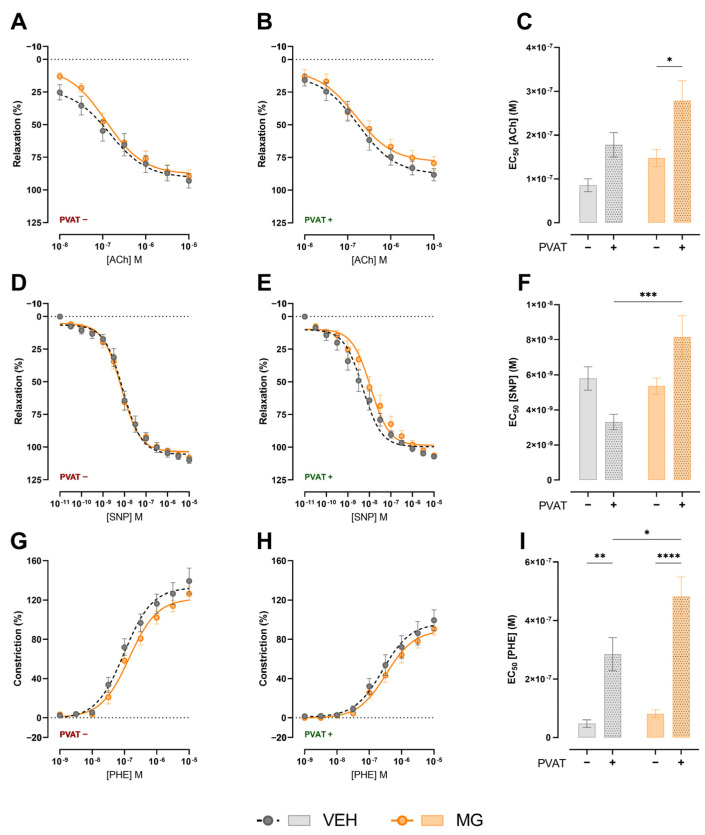
Effects of early exposure to methylglyoxal during lactation on vasomotricity. During the vasomotricity experiments, MG animals presented similar responses to the VEH group in the absence of PVAT for ACh (**A**,**C**) or SNP (**D**,**F**) mediated relaxation, and in PHE (**G**,**I**) mediated constriction. However, in the presence of PVAT, the responses were altered in the vessels of MG animals for ACh (**B**,**C**) or SNP (**E**,**F**) mediated relaxation and in PHE (**H**,**I**) mediated constriction. Two-way ANOVA followed by Sidak’s post hoc test was used for the analysis of the influence of PVAT on vasomotricity. * *p* < 0.05, ** *p* < 0.01, *** *p* < 0.001, **** *p* < 0.0001 vs. VEH.

We sought to evaluate vasorelaxation and vasoconstriction in the presence or the absent of PVAT. In PVAT^−^ preparations, the results were similar for endothelium-mediated relaxation (Figure 3A,C), and NO induced smooth muscle relaxation (Figure 3D,F). However when PVAT was present in the preparation, despite visual similarity between the curves, EC_50_ was different in endothelium-mediated relaxation (Figure 3B,C; VEH 1.778 × 10^−7^ ± 2.77 × 10^−8^ [ACh] M vs. MG 2.784 × 10^−7^ ± 4.58 × 10^−8^ [ACh] M; *p* < 0.05) and NO-induced smooth muscle relaxation (Figure 3E,F; VEH 3.310 × 10^−9^ ± 4.42 × 10^−10^ [SNP] M vs. MG 8.156 × 10^−9^ ± 1.21 × 10^−9^ [SNP] M; *p* < 0.05), which suggests the negative effect of PVAT on relaxation responses in MG-exposed offspring.

In opposition, PVAT exerted a negative regulation in the phenylephrine-induced constriction, since PVAT^+^ preparations presented high EC_50_ values in both VEH (Figure 3G–I; VEH PVAT- 4.210 × 10^−8^ ± 1.30 × 10^−8^ [PHE] M vs. VEH PVAT+ 2.845 × 10^−7^ ± 5.66 × 10^−8^ [PHE] M; *p* < 0.05) and MG groups (Figure 3G–I; MG PVAT- 8.083 × 10^−8^ ± 1.41 × 10^−8^ [PHE] M vs. MG PVAT+ 4.820 × 10^−7^ ± 6.66 × 10^−8^ [PHE] M; *p* < 0.05). However, early glycotoxin exposure increased PVAT negative regulation for vasoconstriction (Figure 3I; VEH PVAT+ 2.845 × 10^−7^ ± 5.66 × 10^−8^ [PHE] M vs. MG PVAT + 4.820 × 10^−7^ ± 6.66 × 10^−8^ [PHE] M; *p* < 0.05).

### 3.4. Effects of Early Exposure to Methylglyoxal during Lactation on Cardiac and Aorta Morphology

**Figure 4:**
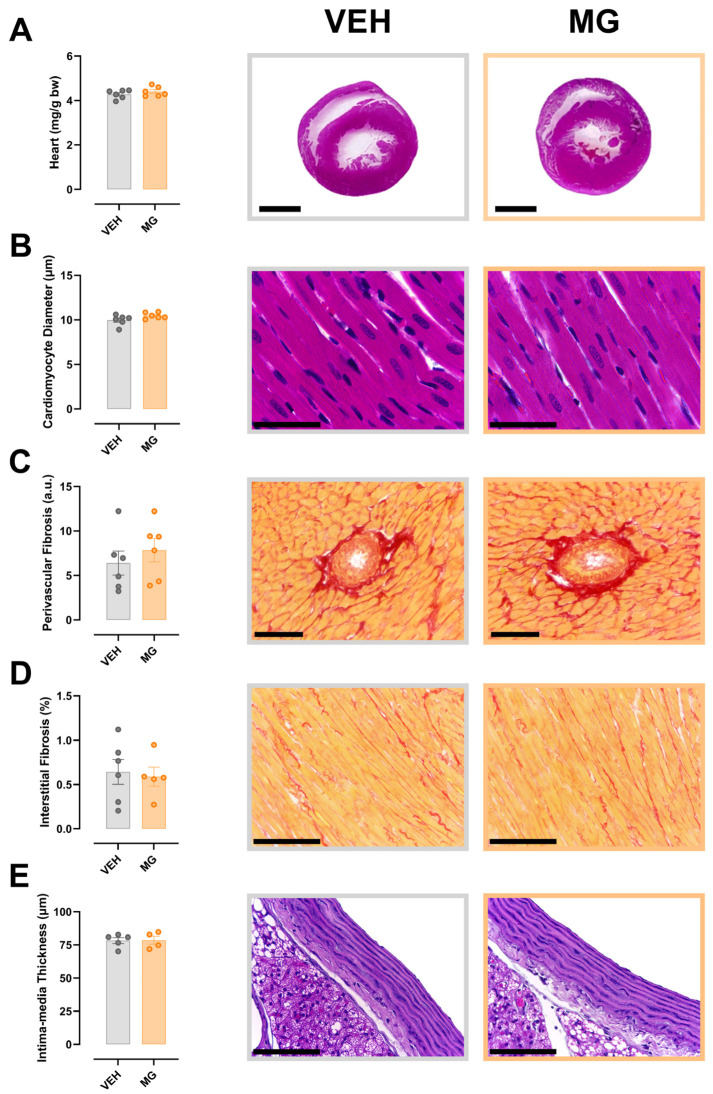
Effects of early exposure to methylglyoxal during lactation on cardiac and aorta morphology. Despite functional impairments, the morphology of MG hearts and aorta remained similar to the hearts of VEH offspring, evidenced by the similar heart weight (**A**), cardiomyocyte diameter (**B**), perivascular (**C**) and interstitial (**D**) fibrosis, and aorta thickness (**E**). Representative images of each parameter are positioned at the right side of the respective graph. Black lines represent, from top to bottom, 2 mm, 25 μm, 40 μm, 100 μm, and 25 μm. Student’s *t*-test was used.

We evaluated the morphology of the heart and aorta of MG offspring. However, no differences were detected in heart weight (Figure 4A), cardiomyocyte diameter (Figure 4B), perivascular (Figure 4C) and interstitial (Figure 4D) collagen deposition, or aorta thickness (Figure 4E).

### 3.5. Effects of Early Exposure to Endogenous Glycotoxins during Lactation on Offspring Development and Vasomotricity

**Figure 5:**
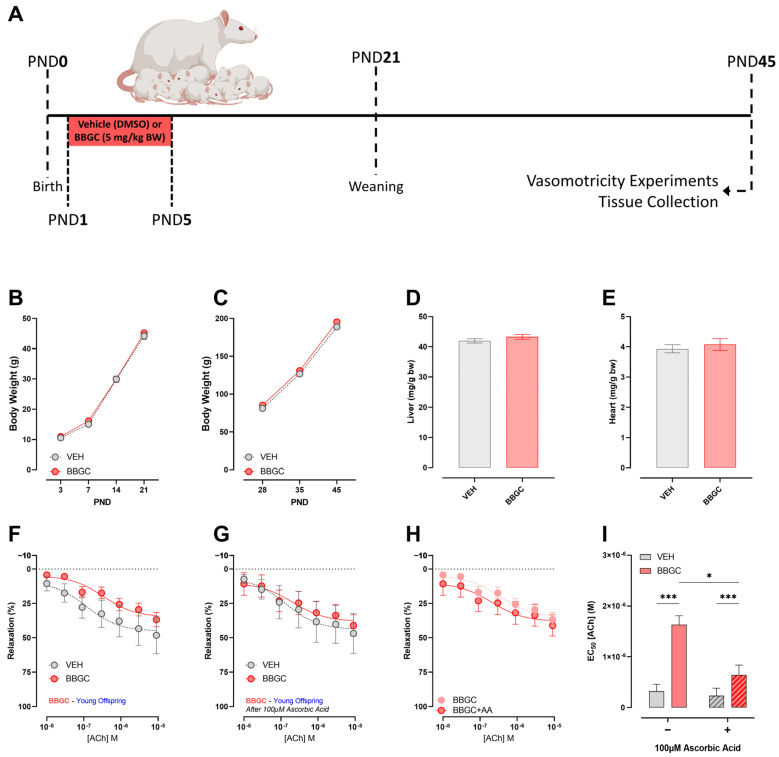
Effects of early exposure to endogenous glycotoxins during lactation on offspring development and vasomotricity. Experimental design (**A**). Body weight of VEH and BBGC offspring remains the same before (**B**) and after weaning (**C**). Early glycotoxin exposure due to maternal GLO-1 inhibition did not affect liver or heart weight (**D**,**E**). No overall differences were observed during the acetylcholine-induced relaxation before (**F**) or after (**G**) ascorbic acid incubation. However, comparing the two curves of BBGC offspring before and after ascorbic acid incubation (**H**), there is an increase in EC_50_ in BBGC offspring both in basal conditions and after the incubation with ascorbic acid (**I**). Student’s *t*-test was used for the analysis of time-independent parameters. Two-way ANOVA followed by Sidak’s post hoc test was used for the analysis of time-dependent parameters and ascorbic acid incubation influence on vasomotricity. * *p* < 0.05 and *** *p* < 0.001 vs. VEH.

Similar to the exposure to methylglyoxal during lactation, the inhibition of GLO-1 in the mothers and consequent endogenous glycotoxin accumulation does not impact body weight gain during lactation (Figure 5B) or after weaning (Figure 5C). Body weight-normalised liver (Figure 5D) and heart (Figure 5E) weights were similar in both groups.

Besides similar dose-responses to ACh before (Figure 5F) and after (Figure 5G) antioxidant incubation, BBGC offspring presented higher values of EC_50_ in both situations when compared to the VEH group, (Before Ascorbic Acid; VEH 1.189 × 10^−7^ ± 1.63 × 10^−7^ M vs. BBGC 3.378 × 10^−7^ ± 2.07 × 10^−7^ M; *p* < 0.05) (After Ascorbic Acid; VEH 1.357 × 10^−7^ ± 2.04 × 10^−7^ M vs. BBGC 2.096 × 10^−7^ ± 2.86 × 10^−7^ M; *p* < 0.05) (Figure 5H,I).

## 4. Discussion

In this study, we aimed to evaluate the cardiovascular effects of early glycotoxin exposure during the lactation period on juvenile rats. We demonstrated that maternal MG exposure during lactation leads to cardiac and vascular dysfunction in the juvenile offspring. Moreover, our results also point to the role of PVAT and impaired antioxidant response on vascular dysfunction caused by early MG exposure. To the best of our knowledge, this study shows for the first time the cardiac functional loss in juvenile rats due to glycotoxin exposure in lactation, which can potentially increase the risk of heart failure at adulthood.

The outcomes of diabetic pregnancies are well described in the literature, leading to LGA offspring [31]. However, little is known about the effects of maternal diabetes during the lactation period [31,32]. In this sense, corroborating experimental data that correlate high circulating glycotoxins and diabetes-induced end-organ damage [33], approaches that address the effects of glycotoxins in the progeny are crucial to understand the phenotype of the newborns exposed to a high-glycotoxin environment [7,25,27]. Mericq and colleagues [27] addressed the effects of maternal diabetes on breast milk and demonstrated that levels of circulating glycotoxins in infants not only correlate with circulating glycotoxins in the mother but also increase with age. In addition, adiponectin levels were negatively correlated with the increased circulating glycotoxins [27].

Here, we chose the dose and route of administration based in previous studies [11,25,34] to avoid interfering with breastfeeding, since the administration of MG intraperitoneally leads to the development of peritonitis and adherence [35]. Also, the oral dose used was shown to result in similar tissue MG concentrations as diabetic rats [36]. Using a similar approach, Francisco and colleagues [25] evaluated the effects of high maternal glycotoxin exposure throughout lactation on the phenotype of the adult offspring. The authors demonstrated that both the plasma and milk composition of MG-injected mothers was rich in fructosamine, an indicator of AGE formation. Also, the plasma of the adult offspring of MG-injected mothers was rich in fructosamine. The main phenotype of the adult offspring of MG-injected mothers at adulthood is insulin resistance and adipose tissue accumulation. In another study, Francisco and colleagues [7] demonstrated that direct exposure to glycotoxins during the same period in previous studies leads to insulin resistance, end-organ oxidative stress, and liver lipid accumulation in adulthood. Also, plasma biochemical parameters, such as fructosamine levels and lipid profile, were not different in MG groups compared to their counterparts. Thus, we hypothesised that metabolic effects of glycotoxin exposure are time-dependent and appear later in life.

Despite the low impact on the metabolic profile, early glycotoxin exposure promotes cardiac dysfunction, leading to lower developed intraventricular pressure, but with no differences in the response to local ischemia. Previous studies have evaluated the effects of glycotoxin exposure on cardiac health over the lifespan [37,38]. Crisóstomo and colleagues [10] demonstrated that chronic administration of MG induces diabetes-like cardiomyopathy in the hearts of Wistar rats and leads to lower AKT activation in ischaemic-reperfusion hearts. On the other hand, the inhibition of AGE circulation through administration of aminoguanidine, a scavenger of reactive carbonyl groups, ameliorates cardiac dysfunction in streptozotocin-induced diabetic mice based on autophagic flux normalization and prevention of ER stress [8]. In the same sense, the reduction in AGE formation through pyridoxamine administration inhibited the effects of MG accumulation in the heart [38]. The same outcome was observed after GLO-1 overexpression in the heart of mice, which preserved post-ischaemia function and morphology [15]. Taken together, the data suggest that the cardiomyocytes of MG-exposed offspring may have metabolic impairments, which impacts the contractile function and could lead to apoptosis later in life.

Together with cardiac alterations, which often induce perceptive symptoms, vascular dysfunction is an important problem for the cardiovascular system, leading to increased peripheral resistance and vascular hypertension due to increased contractile and decreased relaxation responses. Sena and colleagues [11] proved that methylglyoxal increases oxidative stress, attracts immune cells, and promotes endothelial dysfunction in Wistar rats, in addition to worsening the same phenotype in Goto-Kakizaki (GK) rats. MG also promotes increased autophagic flux in several endothelial cell lineages, leading to anti-angiogenic and apoptotic signals, which means low vascularization and increased tissue hypoxia [16]. Another factor that contributes to endothelial dysfunction is perivascular adipose tissue health. PVAT can impact smooth muscle responses to endothelium-derived substances and changes endothelial cell viability through the differential release of adipokines [17,18,39]. Here, we evaluated the endothelium-mediated relaxation using Acetylcholine and relaxation via vascular smooth muscle cells through NO donor Sodium Nitroprusside, both in the presence or absence of PVAT. In juvenile rats, the Ach-mediated relaxation remains the same between the groups in PVAT- aorta rings. In PVAT+ rings, the EC_50_ was increased in both groups in comparison to PVAT- rings but was further increased in the aortas of MG-exposed offspring. In contrast, PVAT+ rings from MG decrease the magnitude of the phenylephrine-induced constriction, evidenced by the raised EC_50_ compared to their counterparts. Despite the effects on PVAT, early endogenous glycotoxin exposure through maternal GLO-1 inhibition lowered ACh responsivity in the thoracic aorta of juvenile BBGC-exposed offspring. Both groups responded to the ex vivo antioxidant exposure, but this response was lower in MG-exposed offspring, suggesting that the redox state was impaired. Azul and colleagues [17] proved that the thoracic aorta PVAT from non-obese type 2 diabetic rats (GK) contributes to endothelial dysfunction through increased macrophage infiltration, proinflammatory cytokine release, and reduction of antioxidant enzymes. The same conditions were observed in aorta from obese type 2 diabetic mice [18].

Given the observed functional impairments, we sought to evaluate the morphological alterations in the heart and aorta due to early glycotoxin exposure. In this study we evaluated the cardiomyocyte diameter, perivascular and interstitial collagen deposition, and aorta wall thickening. None of these parameters were changed in MG offspring, showing that there is a time-dependent component to the progression of cardiovascular disease, which needs to be assessed in further studies. Hypertrophic cardiomyocyte is a marker of diabetic cardiomyopathy; however, the inhibition of AGE formation using aminoguanidine has been shown to inhibit cardiomyocyte hypertrophy in adult animals, revealing the role of glycotoxins in this disease [8]. Regarding inflammation and collagen deposition, Fransisco and colleagues [7] showed that the direct exposure to MG during lactation leads to increased collagen deposition in the liver and kidney of adult rats, in addition to increased oxidative stress in these tissues. These results showed the time-dependent progression of glycotoxin-derived cardiovascular disease, and in puberty the symptoms begin to appear.

Given the observed changes resulting from increased exposure to methylglyoxal during the lactation phase, we hypothesise that increased detoxification of methylglyoxal may be useful in preventing this condition later in life. Complex interventions such as the overexpression of GLO-1 have already been carried out, which improved the detoxification of MG and also ameliorated the outcomes of acute myocardial infarction [9,15]. In addition, we propose the evaluation of the increased MG detoxification through the injections of MG scavengers or GLO-1 activators in the mother or directly in their offspring [38,40,41,42]. Also, increased glycotoxin detoxification can contribute to the reduction of oxidative stress [38,43,44], which can ameliorate observed cardiovascular dysfunction.

## 5. Conclusions

In conclusion, our data suggest that early glycotoxin exposure leads to cardiac and vascular impairments. However, as a limitation of our study, we were unable to assess either the molecular factors by which these effects are caused or treatments that mitigate these effects. Other studies could evaluate whether the observed effects, especially in the heart, are dependent on mitochondrial dysfunction and oxidative stress. The aforementioned studies conducted by Francisco and colleagues [7,25] showed that the effects observed in this study tend to worsen. In this sense, knowing the effects that occur during a phase of phenotypic plasticity as relevant as puberty can open doors to new interventions that can reduce or even abolish the effects that would manifest later in life is of great importance for the studies conducted in the progeny of diabetic mothers.

## Data Availability

The datasets generated during and/or analysed during the current study are available from the corresponding author upon reasonable request.
